# Genetic Overexpression of NR2B Subunit Enhances Social Recognition Memory for Different Strains and Species

**DOI:** 10.1371/journal.pone.0036387

**Published:** 2012-04-27

**Authors:** Stephanie A. Jacobs, Joe Z. Tsien

**Affiliations:** Brain and Behavior Discovery Institute and Department of Neurology, Medical College of Georgia, Georgia Health Sciences University, Augusta, Georgia, United States of America; University of Pittsburgh, United States of America

## Abstract

The ability to learn and remember conspecifics is essential for the establishment and maintenance of social groups. Many animals, including humans, primates and rodents, depend on stable social relationships for survival. Social learning and social recognition have become emerging areas of interest for neuroscientists but are still not well understood. It has been established that several hormones play a role in the modulation of social recognition including estrogen, oxytocin and arginine vasopression. Relatively few studies have investigated how social recognition might be improved or enhanced. In this study, we investigate the role of the NMDA receptor in social recognition memory, specifically the consequences of altering the ratio of the NR2B∶NR2A subunits in the forebrain regions in social behavior. We produced transgenic mice in which the NR2B subunit of the NMDA receptor was overexpressed postnatally in the excitatory neurons of the forebrain areas including the cortex, amygdala and hippocampus. We investigated the ability of both our transgenic animals and their wild-type littermate to learn and remember juvenile conspecifics using both 1-hr and 24-hr memory tests. Our experiments show that the wild-type animals and NR2B transgenic mice preformed similarly in the 1-hr test. However, transgenic mice showed better performances in 24-hr tests of recognizing animals of a different strain or animals of a different species. We conclude that NR2B overexpression in the forebrain enhances social recognition memory for different strains and animal species.

## Introduction

In all species that live in social groups, conspecific recognition is essential for individual and species survival. The ability to recognize another individual as familiar is the basis on which social relationships are founded. In primates, recognition of a conspecific as familiar relies primarily on visual and auditory cues. In rodents, and many other animals, olfactory or pheromonal cues are the primary cues for the development of social memory [1]. Social memory may be useful in maintaining social hierarchies, recognition of offspring, and in pair bonding [2]. Many studies have shown that social recognition undergos modulation by hormones including estrogen, oxytocin, and arginine vassopression. Additionally, the sex of both the subject animal and the stimulus animal are known to influence the strength of the memory formed during a social encounter.

Social memory is an emerging area of interest in memory research. It is not clear as to the cellular mechanisms of controlling the formation of social recognition memory, maximum duration of memory, as well as the brain areas involved in the acquisition and consolidation of social memories. One of the most well-known receptors for the modulation of synaptic plasticity in learning and memory is the *N*-methyl-D-aspartate receptor (NMDAR). NMDARs have been found to be the rate-limiting factor in many forms of associative learning and memory [3], [4], for review see [5]. NMDARs are ionotropic glutamate receptors that usually consist of four subunits, two obligatory NR1 subunits and two NR2A–D subunits. The NR2 subunits are developmentally and regionally regulated within the brain, with the NR2A and NR2B subunits being the main subunits available in excitatory neurons in the forebrain areas, the main areas responsible for learning and memory mechanisms [6], [7]. Our previous work has demonstrated that the NR2B subunit enhances learning and cognition in multiple animal species [4], [8], [9], [10], [11], [12]. NR2B animals have shown enhanced learning in a variety of memory paradigms including object recognition, fear conditioning, fear extinction, Morris Water Maze, and spatial working memory [4], [12]. Previous experiments by another group using these animals have also shown that the NR2B animals have enhanced non-social olfactory memory over their wild-type littermates [13]. In the literature, the role of the NMDA receptors in social recognition has been suggested from pharmacological studies. For example, systemic administration of NMDA has been shown to improve social recognition in rats [14]; while antagonism of the NMDARs impair social recognition at times longer than 30 minutes [15], [16]. Thus, these experiments support the notion that the NMDA receptor is important for social recognition.

In this study, we set out to examine how changes in the NMDA receptor subunit composition may alter social memory and behavior. Specifically, we have investigated the effects of the genetic overexpression of the NR2B subunit in the forebrain regions, on social recognition between an adult male mouse and a juvenile male of the same strain using several paradigms. Furthermore, we also examine the social memories of the NR2B mice for recognizing different strains. Finally, we also examine NR2B mice on their social recognition of a different animal species (rats). Based on these studies, we find that the NR2B animals have enhanced learning and memory abilities, specifically across different strains and animal species.

## Methods

### Animals

Adult male NR2B transgenic animals, and their wild-type littermates, ages 6–9 months, were breed in house from previously generated lines. Animal genotypes were confirmed by PCR analysis of a tail biopsy. Animals were group housed (3–5 per cage) in a standard temperature and humidity controlled animal vivarium with free access to food and water except during social encounters. We used adult male mice aged 6–9 months based on previous studies demonstrating that younger male mice are more aggressive and show more sexually motivated behavior than their older counterparts which masks their recognition abilities [17], [18]. A total of 58 subject mice were used for behavior experiments with groups of animals being rotated through social recognition paradigms allowing for at least two weeks between behavioral testing to prevent any fatiguing effects. Lighting followed a 12∶12 light∶dark cycle with all experiments taking place during the light phase of the cycle. Juvenile male mice, a total of 36 animals, one month of age, were used as stimulus animals to reduce aggression [17], [18]. Juveniles were not reused after each social recognition paradigm. Throughout all experiments, if during the initial encounter the subject animal did not spend at least 25 seconds investigating the juvenile it was removed from the study and the times were not recorded into the data set [19]. The adult male mouse exploring the juvenile was defined by when the adult mouse had its head directed toward the juvenile within 1 cm of the juvenile, or was touching, smelling, or licking the face or anogenital region of the juvenile, or following closely behind (≤1 cm) the juvenile [20]. All behavioral testing occurred in a specially designed noise-reduced, dimly lit animal behavior room.

### Ethics Statement

All protocols were approved by the Institutional Animal Care and Use Committee of the Georgia Health Sciences University, and are in strict adherence with the National Institutes of Health Guide for Care and Use of Laboratory Animals.

### Acute isolation effects of social memory

The first paradigm we used, first described by Thor and Holloway [20], involves obtaining a recognition index by comparing the amount of time the animal spends exploring a juvenile in an initial encounter, with that of the same animal exploring either the identical juvenile or a novel juvenile after 1 hour or 24 hours [21]. The mouse demonstrates a memory of the familiar juvenile mouse by a reduced investigation time in the second encounter. This paradigm is based on the mouse's natural tendency to explore the unfamiliar animal while spending less time exploring an animal that is more familiar.

To assess the effect of acute isolation on social memory we separated group housed animals 24 hours prior to testing into individual cages identical to their original cages, and continued single-housing through the duration of the testing period. During the initial encounter a juvenile male mouse was introduced to the home cage of the adult male for 5 minutes. The amount of time the adult male mouse spent exploring the juvenile was measured.

After the 1-hr or 24-hr interval, either the same juvenile mouse or a novel juvenile mouse was placed back into the home cage of the adult mouse for 5 minutes. The time that the adult mouse spent exploring the juvenile mouse was recorded. A reduction in the amount of time spent exploring the familiar mouse is indicative of the adult mouse remembering the initial encounter with that animal.

### Habituation – Dishabituation social memory

The second paradigm that we employed was the habituation/dishabituation paradigm [22], [23] in which the subject animal is exposed multiple times to the stimulus juvenile animal. The reduced investigation times in subsequent exposures is thought to be a result of the memory of the familiar animal. In our test, we also employed a fifth exposure session of the subject mouse to a novel individual to control for fatigue of the subject to social investigation of the other mouse.

To assess the habituation-dishabituation paradigm in our animals, we separated group housed transgenic animals and their wild-type littermates 15 minutes prior to testing into individual testing cages identical to their original cages. During the initial encounter a juvenile male mouse was introduced to the home cage of the adult male for 1 minute. The amount of time the adult male mouse spent exploring the white juvenile was measured. Exploration criteria used was the same as previously stated. After one minute the juvenile mouse was removed from the testing cage and placed into a holding cage. After a ten minute delay the juvenile mouse was reintroduced into the testing cage with the adult male mouse for the second trial. This was repeated for four trials. For the fifth trial, a novel juvenile is introduced into the testing cage with the adult male. This trial is used as a control to demonstrate that the reduction in investigation is not due to fatigue or habituation.

### Interstrain social memory

To assess the effect of a different color and strain of the stimulus mouse on social memory, we separated group housed transgenic animals and their wild-type littermates (B6× CBA strain) 15 minutes prior to testing into individual testing cages identical to their original cages. During the initial encounter a white juvenile male mouse (BALB/c) was introduced to the home cage of the adult male for 5 minutes. The amount of time the adult male mouse spent exploring the white juvenile was measured. Exploration criteria used was the same as previously stated. At the conclusion of the training the animals used for the 24 hour recall testing were returned to their home cage with their littermates. Animals used for one hour recall training remained in their testing cages for timing reasons.

After 1-hr or 24-hr intervals, either the same juvenile white mouse (BALB/c) or a novel white juvenile mouse (BALB/c) was placed back into the testing cage of the adult mouse for 5 minutes. The time that the adult mouse spent exploring the juvenile mouse was recorded. A reduction in the amount of time spent exploring the familiar mouse is indicative of the adult mouse remembering the initial encounter with that animal.

### Social memory across different species

We also wanted to investigate the social memory between distinct rodent species. We tested the memory of the transgenic NR2B mice and their wild-type littermates for juvenile male rat counterparts (Long Evans). To protect our transgenic animals from previously noted muricide [24], we constructed a mesh chamber to enclose the rat while allowing for access to all four sides and the top of the chamber. The mice were allowed to explore the empty chamber for 10 minutes the day before the testing. Transgenic mice and their wild-type littermates were separated into large clean rat cages 30 minutes prior to testing and allowed to acclimate to the testing cage. The rats were placed into the chamber and the chamber was placed into the testing cage for five minutes. During the five minutes, the time that the mice spent exploring the rat chamber was measured. The mouse was said to be exploring the chamber if the mouse's nose was directed toward any surface of the chamber within 1 cm of the chamber. Simply touching the chamber was not counted based on the natural tendency for the mouse to climb onto surfaces to explore what is on top of or above it. Animals used for one hour recall training remained in their testing cages for timing reasons.

After the 1-hr or 24-hr interval, either the same rat or a novel rat was placed back into the chamber, and the chamber was placed back into the testing cage with the adult mouse for 5 minutes. The time that the adult mouse spent exploring the chamber was recorded. A reduction in the amount of time spent exploring the rat chamber is indicative of the adult mouse remembering the initial encounter with that animal.

### Statistical analysis

All data are expressed as mean ± SEM. Student T-tests were used to analyze significance of initial exploration versus recall exploration sessions. Differences between groups were analyzed using one-way ANOVA with Tukey-Kramer analysis. Significance level was p<0.05.

## Results

To study social memory in mice, we have used two paradigms of social recognition behaviors. In the first paradigm, an exploratory preference was obtained by comparing the amount of time the subject animals spent exploring the stimulus animal in the second trial with that of the first. A reduction in the amount of time spent exploring a familiar animal in the second exposure was interpreted as the recognition of that animal. In the second paradigm, the subject animal was exposed to a stimulus animal several times within a short period of time. The reduction of investigation times in each subsequent encounter was interpreted as a recognition memory of the stimulus animal.

### Social memory assessment with and without isolation

#### Short-term social recognition memory

**Figure 1:**
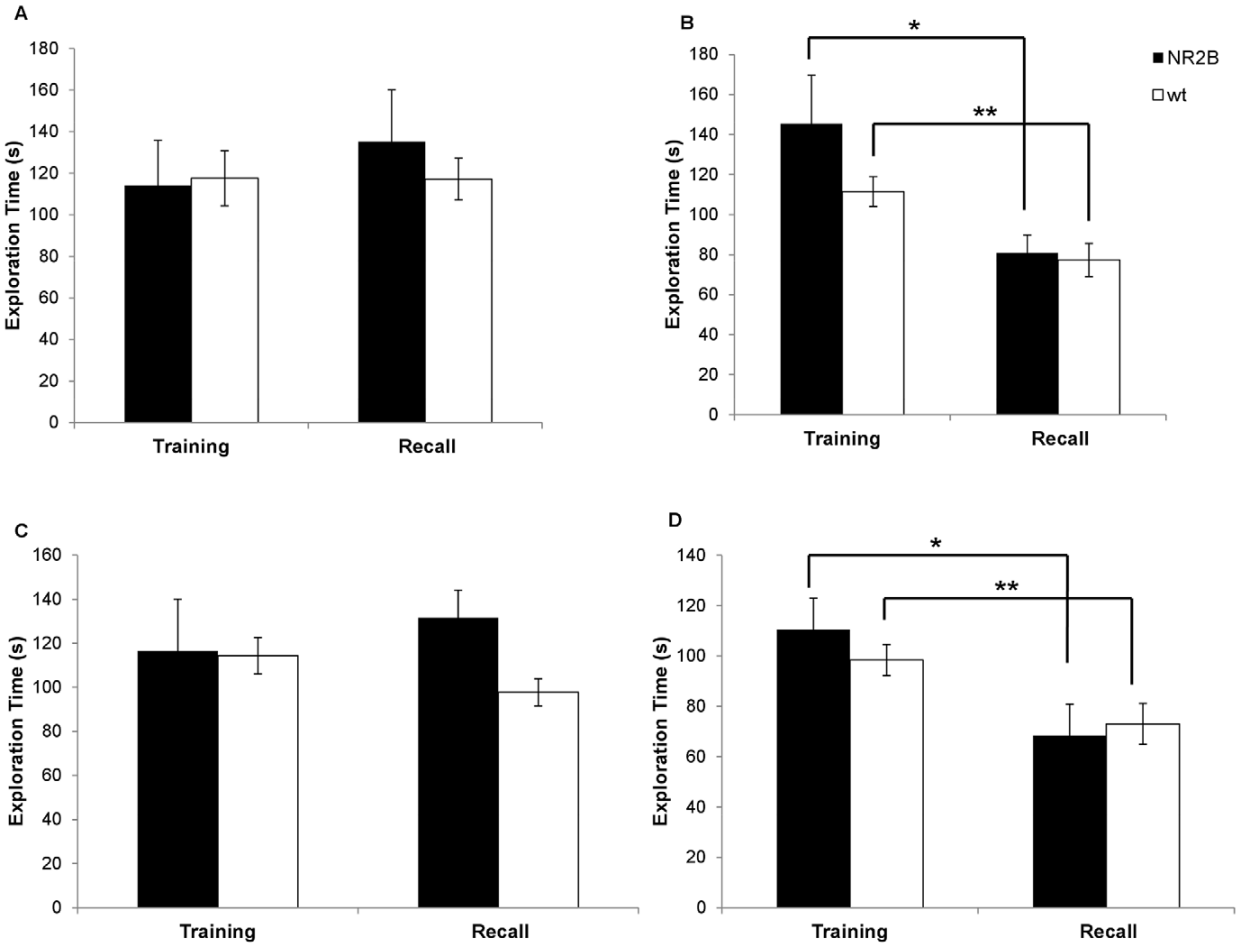
1-hr and 24-hr recall with no prior isolation. A. Initial encounter and one hour recall using a novel mouse. Here this is no reduction in the investigation time during the second encounter. B. Both the NR2B and the wild-type animals demonstrate a significant reduction in investigation times of the familiar juvenile indicating a memory of that animal. (* p = 0.037, ** p = 0.005) C. There were no significant differences seen between the investigation times in the initial encounter and the second encounter 24 hours later when a novel animal was used at the second encounter. D. Both groups of animals spent significantly less time investigating the familiar animal at the 24 hour recall session. (* p = 0.045, ** p = 0.02).

In order to test the social memory in our transgenic animals, we first tested them without any prior isolation from their cage mates. Each animal was separated into a testing cage identical to their home cage and exposed to the stimulus animals for two 5 minute encounter session separated by 1 hour. In the one hour delay test, when a different juvenile was used in the second encounter session both groups had similar initial investigation times (NR2B: n = 5, 144.2±21.60 s; Wt: n = 15, 117.59±13.21 s; Fig. 1A) as well as similar times in the second encounter session with a novel juvenile (NR2B: 135.22±24.86 s; Wt: 117.17±10.05 s). There were no significant differences found between the first and second encounters in either of the groups (NR2B: p = 0.54; Wt: p = 0.98).

We then tested the animals using the identical animal in the second encounter, one hour after the first encounter. In this experiment, there were no significant differences in exploration times between the groups in the initial encounter, demonstrating that both groups had similar motivation and interest in the juveniles (NR2B: n = 5, 145.46±24.22 s; Wt: n = 14, 111.52±7.46 s; Fig. 1B). In the second encounter one hour later, both groups had a significant reduction in exploration times (NR2B: 80.79±9.07 s, p = 0.037; Wt: 77.33±8.26 s, p = 0.005). This shows that all animals tested are able to form short-term social recognition memory.

**Figure 2:**
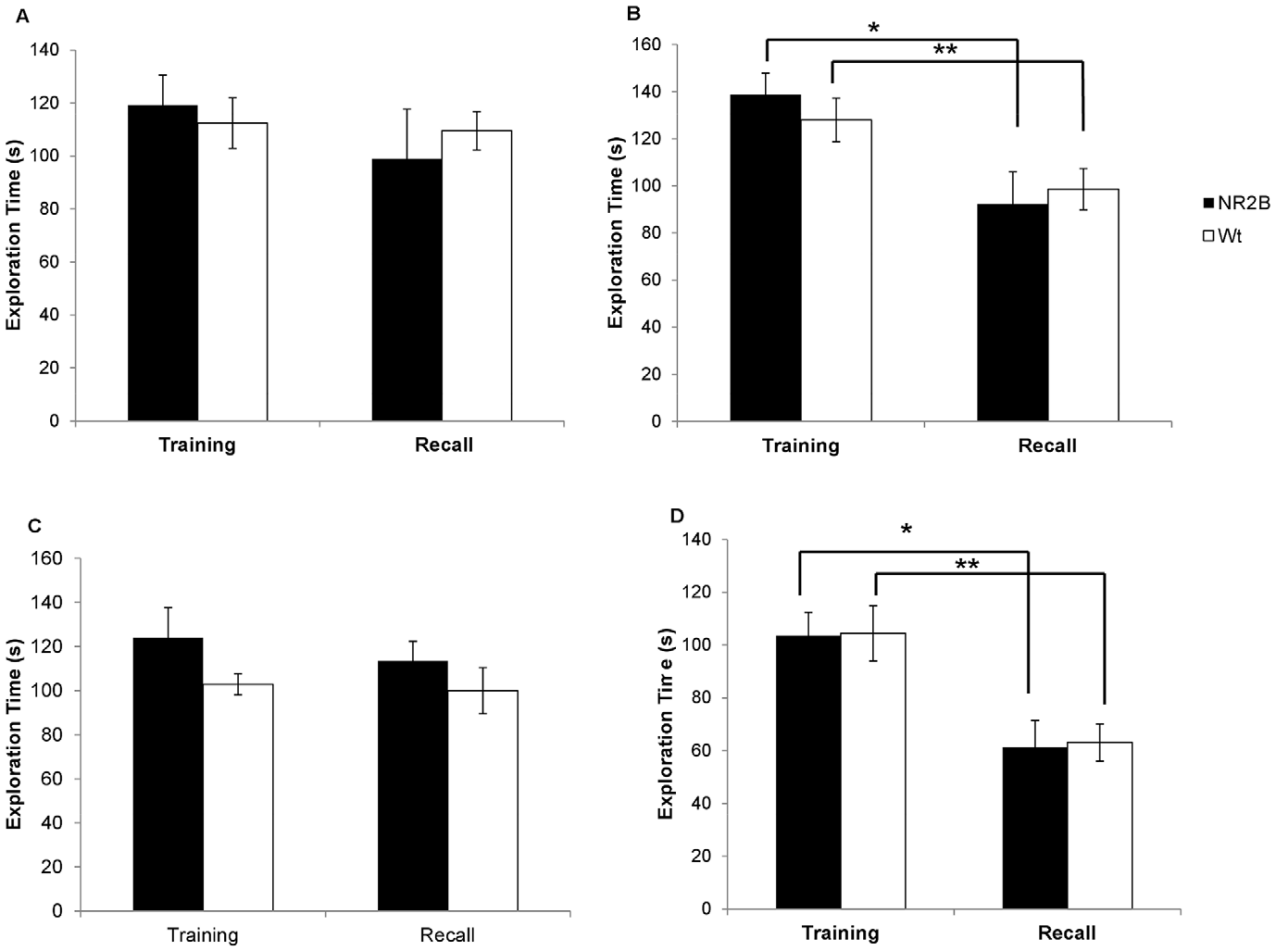
1-hr and 24-hr recall after 24-hr social isolation. A. No reduction in investigation times was seen in the second encounter with a novel animal after one hour. B. Initial encounter and one hour recall using familiar mouse. Here a significant reduction in the investigation times is seen in both the NR2B and the wildtype animals (* p = 0.018, ** p = 0.029) C. There was no reduction in investigation time at the 24 hour recall encounter using a novel mouse. D. A significant reduction was seen in the investigation times of the familiar mouse in both groups after 24 hours. (* p = 0.0136, ** p = 0.001).

We then tested the short-term social memory in our transgenic animals by using a 24 hour isolation protocol. We separated the mice into single cages for a period of 24 hours immediately before testing and continuing throughout the duration of the test to determine if acute isolation would alter their social behavior. For the testing period the subject animal was exposed to the stimulus juvenile animals for two 5 minute encounter sessions separated by 1 hour. In the first session, both groups had similar initial investigation times (NR2B: n = 5, 119.27±11.26 s; Wt: n = 15, 112.45±9.60 s, Fig. 2A). The groups also had similar investigation times in the second encounter session with the novel juvenile (NR2B: 98.99±18.73 s; Wt: 109.55±7.27 s). There were no significant differences between the first and second encounters in either of the groups (NR2B: p = 0.38; Wt: p = 0.81).

We then tested the animals using the identical animal in the second encounter one hour after the first encounter. Again, there were no significant differences in exploration times in the initial encounter, demonstrating that both groups had similar motivation and interest in the juveniles (NR2B: n = 5, 138.71±9.16 s; Wt: n = 15, 127.95±9.26 s, Fig. 2B). In the second encounter one hour later, both groups showed significant reductions in the exploration time of the familiar juvenile (NR2B: 92.24±13.68 s, p = 0.02; Wt: 98.59±8.74 s, p = 0.03). These results indict that relatively short periods of isolation do not significantly affect the short-term social memory in our transgenic animals or their wild-type littermates.

#### Long-term social recognition memory

It has been suggested that socially grouped mice also exhibit long-term social recognition memory. Thus, we examined the long-term social recognition memory of our animals, again using group-housed animals and acute isolation protocols. We first tested long-term social memory of group housed NR2B mice and their wild-type littermates. When we tested the animals using different stimulus juveniles in both encounter sessions, we found no significant differences between the groups in the initial encounter (NR2B: n = 5, 116.62±12.99 s; Wt: n = 15, 114.36±8.62 s, Fig. 1C) or the second encounter (NR2B: 131.59±23.44 s; Wt: n = 15, 97.76±8.27 s). We also found no significant reduction in investigation times between the first and second encounters in either of the groups (NR2B: p = 0.59; Wt: p = 0.176).

We then tested our transgenic animals using the identical stimulus juvenile in both encounters. In this experiment, NR2B animals and their wild-type littermates exhibited similar investigation times in the initial encounter (NR2B: n = 5, 110.43±12.53 s; Wt: n = 14, 98.44±6.16 s, Fig. 1D) demonstrating similar interest and motivation in exploring the unfamiliar juvenile. In the subsequent 24 hour retention test, the NR2B and the wild-type animals both demonstrated a significant reduction in the investigation time of the familiar juvenile (NR2B: 68.42±12.44 s, p = 0.045; Wt: 73.04±8.14 s, p = 0.02).

We performed another experiment by using an acute isolation protocol. We separated the mice into single cages for a period of 24 hours prior to a social interaction training session. Again, animals remained isolated through the duration of the retention tests. When the two different stimulus juveniles were used for each encounter, the acute isolation had no effect on either the initial encounter investigation times (NR2B: n = 5, 123.99±17.42 s; Wt: n = 15, 102.86±8.09 s, Fig. 2C) or the second encounter investigation times (NR2B: 113.45±13.61 s, p = 0.65; Wt: 100.02±4.84 s, p = 0.88). No significant differences were seen between the groups in either encounter.

When tested using identical animals for both the initial encounter and the second encounter, the NR2B animals and their wild-type littermates had similar initial investigation times (NR2B: n = 5, 103.61±8.82 s; Wt: n = 15, 104.46±10.43 s, Fig. 2D). Both the NR2B and the wild-type groups showed significant reductions in their mean investigation times in the second encounter (NR2B: 61.29±10.14 s, p = 0.01; Wt: 63.10±7.04 s, p = 0.001), indicating their comparable ability to form long-term social memories even after an isolation period of 24 hours.

### Habituation – Dishabituation social behaviors

**Figure 3:**
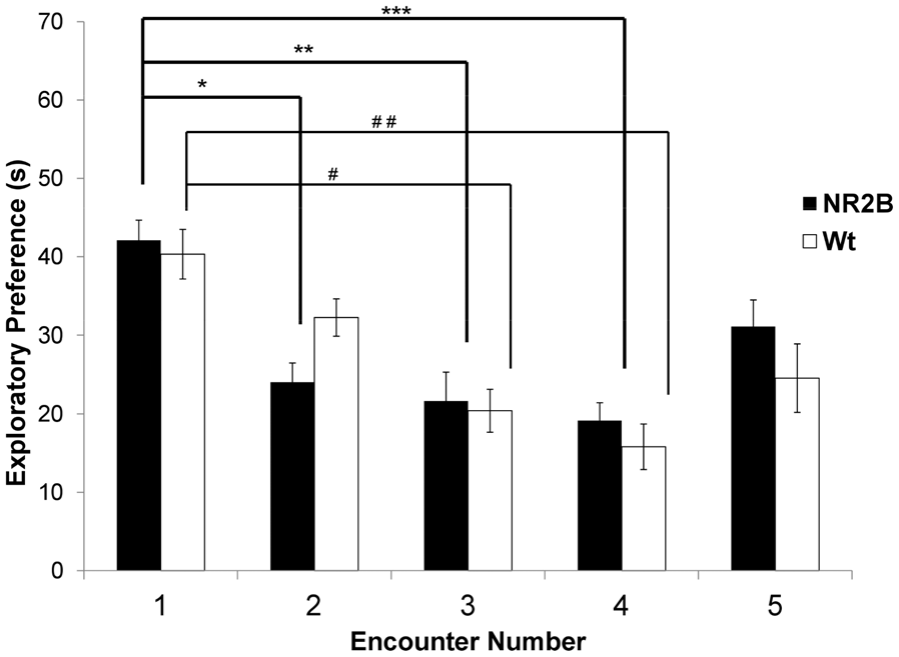
Habituation-Dishabituation Paradigm. During this paradigm the animals were exposed to the stimulus mouse for one minute for four sessions separated by a 10 minute rest period. The NR2B animal showed a significant reduction in exploration times at the second encounter which continued to the fourth encounter. The wild-type animals showed a significant reduction in investigation time from the initial exposure starting at the 3 exposure. During the fifth exposure session, a novel animal was used as a control to account for fatigue and disinterest as a result of repeated exposures. (* = 4.28×10^−5^, ** = 1.6×10^−4^, *** = 6.3×10^−4^; # = 7.7×10^−5^, # # = 7.1×10^−6^).

We also wanted to test our animals in another paradigm of social recognition in which a familiar juvenile is presented to the subject mouse multiple times for a small amount of time (1 minute) with a short delay (10 minutes) between exploration sessions. This paradigm is a more ecological approach to examining social behavior by mimicking the animals natural social encounters [25]. All groups displayed similar investigatory behavior in their initial encounter (Fig. 3). The NR2B group showed significant decreases in investigation times in the second session and maintained significantly shorter investigation times in the third and forth encounters as well. The wild-type group showed decreased investigation times in the second encounter which reached significance during the third encounter. During the dishabituation encounter with a novel juvenile, neither of the groups reached the same level of investigation as in the initial encounter. In this paradigm, the NR2B animals were able to form a more robust memory of the juvenile quicker than the wild-type animal demonstrating their enhanced cognitive abilities.

### Interstrain social recognition memory

#### Short-term social memory of an interstrain conspecific

**Figure 4:**
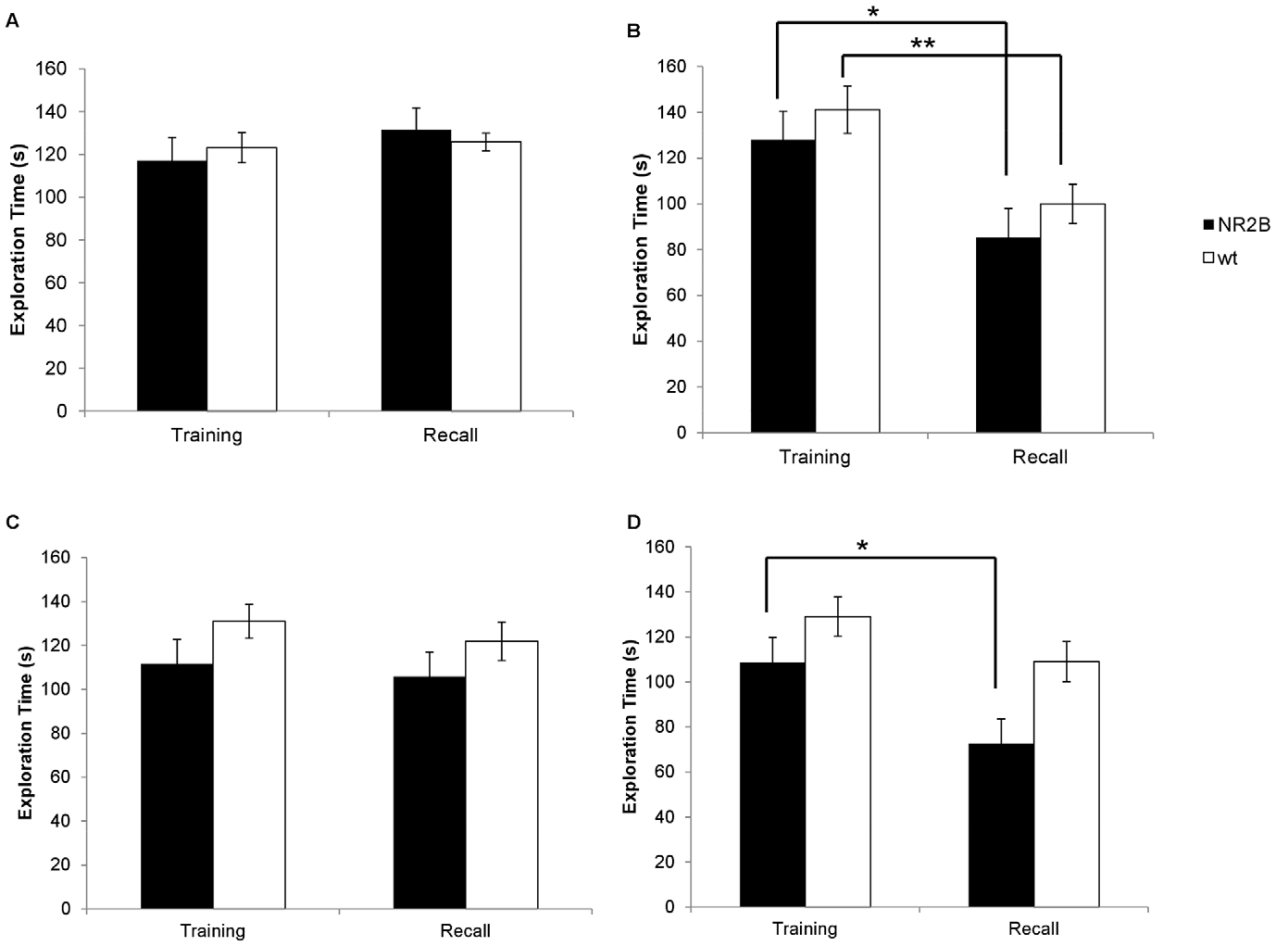
Interstrain social recognition at 1-hr and 24-hr using a white mouse. A. No significant differences were found during the second exposure using a novel mouse after one hour. B. After one hour, both the NR2B and the wild-type animals showed a significant reduction in investigation times of the same white mouse that presented in the first encounter. (* p = 0.044, ** p = 0.005) C. During the 24 recall the NR2B animals and the wild-type animals show no reduction in investigation time when a novel mouse is presented in the second encounter. D. After 24 hours only the NR2B animals show a significant reduction in investigation times of the familiar white mouse indicating that the wild-type animal was unable to form a long-term social recognition memory of the white mouse . (* p = 0.048).

While both transgenic and wild-type mice exhibited the similar social recognition memory within the same strain, we wondered if transgenic overexpression of NR2B may have any effect on the animal's ability to recognize mice from different strains. Our transgenic mice and their littermates are bred on a B6× CBA background (black or agouti), accordingly, we used to BALB/c juvenile mice as stimulus mice. We first examined short-term (1 hour) social memory. We found that when using different white juveniles for the first and second encounter, no significant differences were found between the first session (NR2B: n = 5, 117.13±10.81 s; Wt: n = 15, 123.15±7.03 s, Fig. 4A) and the second session (NR2B: 131.48±10.27 s, p = 0.36; Wt: 125.83±4.13 s, p = 0.75).

Next we tested the investigation times when using the identical white juvenile mouse for both sessions. Both groups spent similar amounts of time during the initial encounter investigating the white juvenile (NR2B: n = 5, 128.02±12.46 s; Wt: n = 15, 141.2±10.32 s, Fig. 4B). At the second exposure, both groups of animals spent significantly less time exploring the familiar white mouse (NR2B: 85.3±12.78 s, p = 0.04; Wt: 100.00±8.62 s, p = 0.005).

#### Long-term social recognition memory of an interstrain conspecific

We then tested the long-term social recognition memory of our mice using the 24 hour retention test. When different mice were used for each encounter, the investigation times for the first encounter (NR2B: n = 5, 111.73±11.78 s; Wt: n = 14, 131.02±8.59 s, Fig. 4C) and the second encounter (NR2B: 105.87±11.05 s; Wt: 121.9±7.67 s) did not significantly vary (NR2B: p = 0.73; Wt: p = 0.43).

Interestingly, when using the same BLAB/c juvenile as the stimulus mouse, the NR2B animals showed significant reductions in the investigation times in the second encounter (NR2B: n = 5, 72.66±10.97 s, Fig. 4D) as compared to the first encounter (NR2B: 108.73±11.08 s, p = 0.048). On the contrary, their wild type littermates showed no significant decrease in the investigation times between the first (Wt: n = 15, 129.07±8.76 s) and second encounters (Wt: 109.08±8.88 s, p = 0.12). This suggests that the NR2B animals formed robust long-term social recognition memories for the stimulus juvenile animal from a different strain.

### Social memory across species

In order to test the ability of our transgenic animals to form memories of animal outside of their mouse species, we further examined NR2B mice by using a paradigm in which they were exposed to juvenile Long Evans rats. The rat was maintained in a mesh covered chamber to protect the mice. The chamber was placed inside the mouse's testing cage for five minutes in each session.

#### Short-term social recognition across species

**Figure 5:**
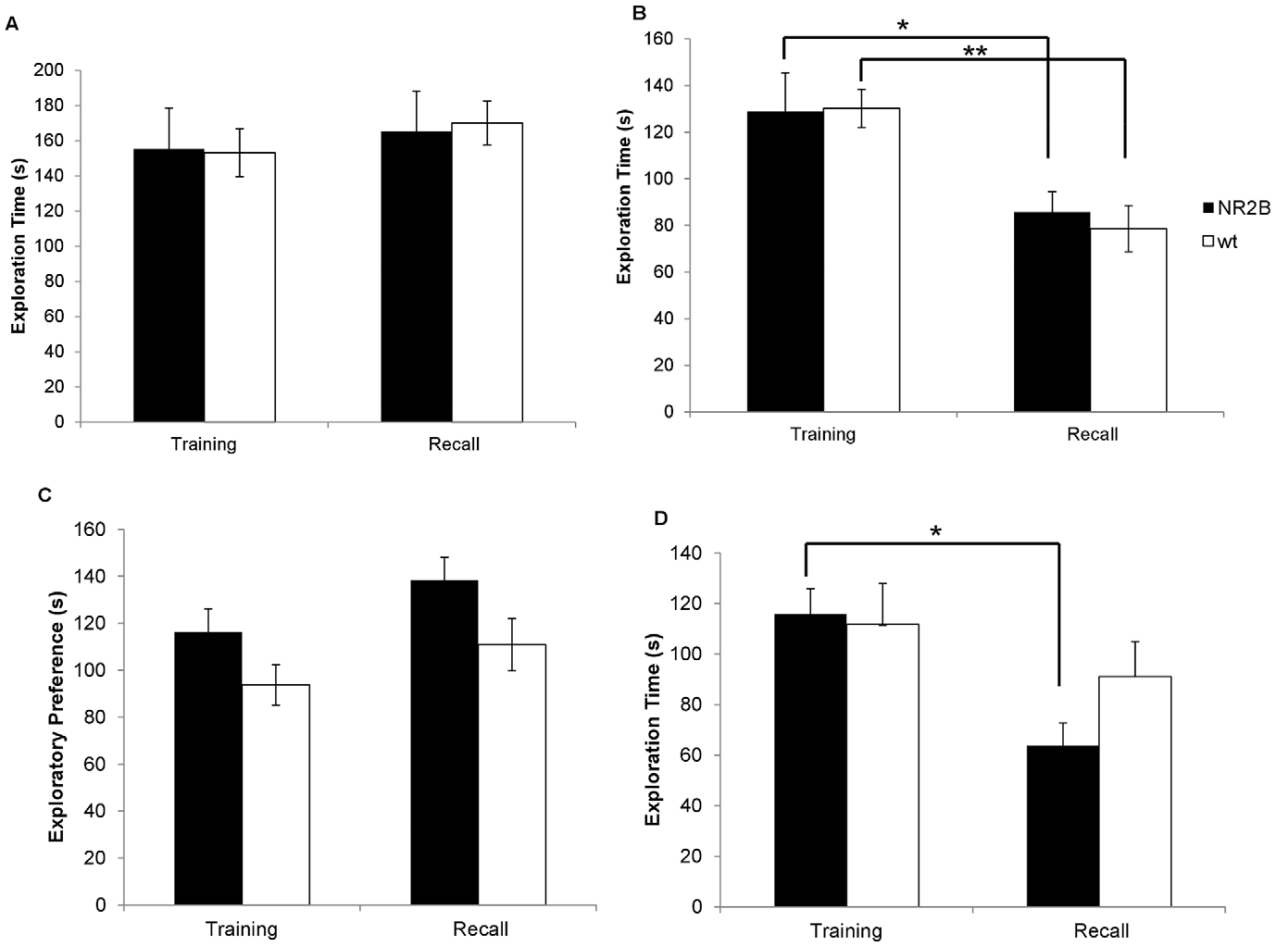
1-hr and 24-hr cross species social recognition. A. In comparison to the initial encounter, neither the NR2B nor the wild-type animals show any reduction in the investigation time of the rats during the second encounter. B. In the one hour recall session with the same rat, both the NR2B and the wildtype animals show a significant reduction in the investigation times indicating that both groups of animals have formed a memory of the familiar rat. (* p = 0.033, ** p = 0.0006) C. There were no significant differences found between the investigation times of the novel rat in the first encounter with the novel rat in the second encounter. D. During the 24 recall session only the NR2B mice showed a reduction in the investigation times of the familiar rat. (* p = 0.001) This indicates that only the NR2B mice were able to form a long-term memory of the rats.

When different rats were used in each encounter, there was no significant difference in investigation times between the first encounter (NR2B: n = 9, 155.22±23.37 s; Wt: n = 12, 153.20±13.55 s, Fig. 5A) and the second encounter (NR2B: 165.36±22.92 s, p = 0.67; Wt: 170.08±12.44 s, p = 0.37). Interestingly, both the NR2B and wild-type groups showed significant decreases in investigation times between the first (NR2B: n = 11, 128.81±16.62 s; Wt: n = 12, 13.16±8.24 s, Fig. 5B) and second encounters (NR2B: 85.72±8.77 s, p = 0.03; Wt: 78.59±9.92 s, p = 0.0006). This demonstrates that both groups are able to form short-term social memories of animals from other species.

#### Long-term social recognition memory across species

After 24 hours all groups showed similar investigation times in both the first social encounter (NR2B: n = 109, 116.29±9.87 s; Wt: n = 12, 93.77±8.59 s, Fig. 5C) and the second encounter (NR2B: 138.45±9.70 s, p = 0.13; Wt: 110.91±11.09 s, p = 0.23) when different rats were used for each encounter. Interestingly, when the same rat was used for both encounters, only the NR2B transgenic animals demonstrated any memory of the rat as seen in the significant reduction in the investigation times from the first encounter (NR2B: n = 11, 115.84±10.02 s, Fig. 5D) and the second encounter (NR2B: 63.67±9.13 s, p = 0.001). The wild-type groups had no reduction in the investigation times from the first encounter (Wt: n = 7, 111.77±16.18 s) to the second encounter (Wt: 91.10±13.87 s, p = 0.35). This also suggests that the NR2B animals are capable of forming long-term social memories of animals from other species, while their wild-type littermates are not.

## Discussion

In our study, we investigated the cellular mechanisms behind the formation of social memories using Tg-NR2B mice in which NR2B is overexpressed in the forebrain regions. This allowed us to explore the effect of a genetic manipulation of the NR2B∶NR2A subunit ratio of the NMDA receptor on social memory. We have shown previously that our NR2B animals have enhanced learning and memory compared to their wild-type littermates in multiple forms of memory including recognition memory, avoidance memory and emotional fear memory, spatial memory, and spatial working memory, [3], [4], [8], [9], [12], [13], [26]. The NMDA receptor is thought to be the rate-limiting molecule in synaptic plasticity and the main coincidence detector in the brain. The NMDA receptor has been found to be essential in the acquisition of certain types of memories including spatial memory [27], [28], spatial representation [27], [28], [29], and temporal memory [30]. It is necessary for the consolidation and storage of memory traces into long-term memories [31], [32], [33], as well as the maintenance of remote memories [34]. The NR2B subunit was strategically chosen for upregulation for several reasons. First, the NR2A and NR2B subunits are highly expressed in the forebrain regions. Moreover, the NR2B subunit is relatively higher in the juvenile brain after birth when synaptic plasticity is very high [7], [35], in comparison to adulthood. As the brain ages, a decrease in the NR2B∶NR2A ratio parallels a decrease in synaptic plasticity [36], [37] and memory performance in multiple species beginning at the onset of sexual maturation [38], [39]. This suggests the idea that a higher NR2B∶NR2A ratio improves synaptic plasticity, memory and cognition. Furthermore, the incorporation of the NR2B subunit in the NMDA receptor complex increases the opening duration of the NMDA receptor prolonging the available time for coincidence detection [7]. The NR2B subunit can also be endogenously upregulated, and is significantly increased following environmental enrichment [12]. Surprisingly, the genetic overexpression occluded this increase in NR2B transgenic animals suggesting the enhancements seen in the expression of NR2B are by similar mechanisms [12]. Our results in this study, in conjunction with previous studies, find that the NR2B subunit is able to enhance memory and cognition in multiple forms of memory, including emotional memory, associative memory, recognition memory, and social memory, despite the involvement of different memory mechanisms.

Because social recognition is crucial for species survival, the maintenance of the established social hierarchy and offspring recognition, it is a good representation of an ethologically relevant task in mice [40]. For this reason, we chose to test the ability of our transgenic animals to recognize juvenile conspecifics after one hour and 24 hours. Studies have shown that long-term social memory requires two separate stages of protein synthesis in order to be retrieved at a later time [19], [41]. Indeed, protein synthesis has long been known to be essential for the formation of long-term memory and has been confirmed as necessary in social memory as well [19], [41].

While the olfactory system has been used for many years to test associative or discriminative memory in rodents, the brain regions involved in social recognition are still being investigated, as some studies give differing results in involved brain regions based on lesion studies and c-Fos activation studies [41], [42], [43], [44], [45], [46]. Many studies indicate the involvement of prefrontal cortex, the hippocampus, the olfactory bulb and the amygdala may be involvement in various aspects of social behaviors. Multiple studies show increased c-Fos activation in the medial preoptic area, the medial amygdala and the piriform cortex, [41], [42], [43], [44], [47]. Initial studies indicated that excitotoxic lesions of the dorsal and ventral hippocampi did not impair immediate or 30 minute delay social memory in mice [45], [46], [47]. However, the hippocampus is known to play a key role in human social recognition [48]. Moreover, recent lesion studies have indicated that the hippocampus is involved in the formation of long-term social recognition memory in mice, as well as, immediate social memory [19]. A recently published paper reports an intriguing finding that targeted complete lesions of the hippocampus in which surrounding fibers were spared, have no effect on social recognition at recall times of 5 min, 20 min, 1 hour, 24 hours or 48 hours , whereas targeted lesions of the perirhinal cortex, centered around the mid-posterior rhinal sulcus, greatly impair social recognition in rats at times greater than one hour [49]. The results of the c-fos and lesion studies are fascinating based on findings that show an increase in c-fos activation after hippocampal-dependent learning tasks [50], [51], [52], [53]. Some studies have also found that the brain regions that are activated during a social encounter also depend on the sex of the stimulus animals. Several studies have demonstrated that both the accessory olfactory bulb, and the main olfactory bulb were activated after the subject mouse was exposed to a male mouse, but not after exposure to a female mouse [41], [42], [44]. The amygdala is known to be involved in many forms of emotional memory in addition to recent discoveries of its role in social recognition. Not only is c-fos activation clearly increased in the amygdala, and has been investigated as the central location for estrogen and oxytocin modulation of social learning. Mice lacking estrogen receptors or oxytocin receptors have been found to be impaired in social recognition tasks [54], [55], [56], [57]. These data, as well as other studies, indicate the importance of both the hypothalamus and the lateral septum in social recognition. Our animal model overexpresses the NR2B subunits in the forebrain regions including the olfactory bulb, the amygdala, the cortex and the hippocampus [4], [9], [12], many of the regions thought to be involved in social recognition mechanisms. By examining the involvement of the NR2 subunits in multiple memory mechanisms [24], [58], [59], we can better understand the function of the NR2 subunits and thus the impact of their dysfunction.

Our present study is consistent with the notion that NMDA receptor is important for social recognition memory, and furthermore, provides the genetic evidence that NR2B overexpression can lead to improvement in certain aspects of social memory. In our study, the short-term memory of these NR2B animals was unaffected by acute separation. This finding is in agreement with other studies that have explored the effects of isolation on social memory [19] as well as earlier studies in which the animals were routinely single-housed [20], [22], [60], [61]. In these studies, wild-type animals were fully able to form short-term social recognition memories for up to 2 hours. On the other hand, evidence in literature consistently shows that group-housing is known to improve social memory. Thus, our NR2B and wild-type animals' ability to form long-term social memories of conspecifics when group-housed, is also in agreement with several other studies which demonstrate the ability of the mouse to form long-term social memories [19], [44], [62], [63]. Mice have been shown to discriminate between inbred genetically identical littermates alluding to a distinctive individual odor for each animal [19], [64], [65]. Interestingly, our mice did show long-term social recognition of a familiar conspecific even after an acute isolation period. Previous studies have shown that after 24 hours of isolation, or chronic isolation, there is a significant long-term social memory impairment in rodents, not only in social recognition memory [19], [20], [60], [61] but also expanding to other social behaviors [66] and cognitive tasks [66], [67], [68]. Indeed, in many ways social interaction is a form of enrichment. Social learning is a key tool used by many species to pass down acquired knowledge from generation to generation, such as the songs of songbirds, or hunting techniques used by orcas. The ability of animals to pass down learned knowledge to their young is one of the many advantages of living in social groups. In the lab, enrichment often occurs by allowing animals time to explore larger cages with different toys and social partners. This has been found to improve multiple forms of learning and memory including spatial memory, emotional memory and recognition memory [69], [70], [71], [72], [73], [74]. On the cellular level, environmental enrichment has been found to increase dendritic spine density and to rescue genetic learning deficit [12], [74]. Prolonged social housing may have attributed in the lack of impairment in social recognition seen after only 24 hour isolation of our animals.

In the habituation-dishabituation paradigm our animals, we found that investigatory behavior did not return to the first novel exploratory session as in other studies. It has been suggested that other factors may influence such behaviors this type of paradigm [56]. In the dishabituation trial the subject animals did spend noticeably more time investigating the novel animal than the familiar animal in the previous trial. Interestingly, our NR2B animals showed a significant decrease in the investigation time as soon as the second trial. This indicates that the NR2B animals are able to form a more robust memory of a juvenile conspecific even after a very brief encounter.

Interestingly, little data in the literature shows the influence of the strain of the stimulus mouse on the ability of the subject mouse to form social recognition memories of the stimulus mouse. Moreover, many studies focus on male-female interactions [75], which we did not investigate in this study, but will be of great interest for us to investigate in the future. At present, we used adult males from the background we were investigating, with mice of a different fur color and a different genetic background. Only our NR2B animals are able to remember the familiar conspecific of a different color and strain for 24 hours. This behavior may have natural origins in that feral mice are more likely to mate with members of their own strain [75] which may be beneficial to long-term species survival. Interestingly, wild-type animals have been found to discriminate between novel and familiar females of other strains, whereas oxytocin and oxytocin receptor knockout mice fail to distinguish between females of other strains [75]. Oxytocin infusions are known to rescue this deficit in social recognition [42], [76].

Our NR2B animals and their wild-type littermates show comparable short-term social recognition for the rats, but only the NR2B animals showed a long-term social memory of the rat. Our observations are in agreement with some studies which show that mice can form a social recognition memory of other species [24], [77]. However, it has been shown by Engelmann's group [24] that both the non-volatile and volatile components of a scent signature may be important for the formation of long-term social memory. Due to the risk of muricide to our transgenic animals, we could not allow direct contact between the mice and the rats. The inability to have full access to the body of the subject animals may have, to some extent impaired the long-term social memory in the wild-type animals. Another factor to consider is the stress induced in the mice due to the exposure to the rat. Interestingly, some studies suggest that when a female undergoes a stressful experience just before a social encounter with a juvenile, the female has an enhanced social recognition memory 2 hours later for the juvenile [78]. The exposure to a much larger rat juvenile, even confined to a closed chamber, may have induced stress on the subject mice leading to an increase in their social memory of the juvenile rat. In fact, genetic overexpression of corticotrophin-releasing factor (CRF) in transgenic mice was also found to improve social recognition [79], further confirming the role of stress in enhanced social memory. These observations may account for some of the increase seen in social learning in the NR2B overexpression animals. In our study, a genetic overexpression of NR2B in the forebrain areas was able to achieve long-term social recognition which is not seen in the wild-type animal. Collectively, this would be beneficial for the NR2B mouse in a natural environment and would promote survival of the animal in appropriately dealing with other species, and promote social interaction with other murine strains.

In conclusion, we have shown that NR2B transgenic animals have exhibited several interesting social recognition phenotypes. While the NR2B animals show similar short-term and long-term memory in recognizing the same strain stimulus mice in comparison to that of the wild-type animals, the NR2B transgenic animals have enhanced long-term memory capabilities when forming social memories across different strains and species.
